# Paediatricians’ perspectives on global health priorities for newborn care in a developing country: a national survey from Nigeria

**DOI:** 10.1186/1472-698X-12-9

**Published:** 2012-07-02

**Authors:** Bolajoko O Olusanya, Chinyere V Ezeaka, Ekundayo K Ajayi-Obe, Mariya Mukhtar-Yola, Gabriel E Ofovwe

**Affiliations:** 1 Healthy Start Initiative, Ikoyi, Lagos, Nigeria; 2 Department of Paediatrics, Lagos University Teaching Hospital, Surulere, Lagos, Nigeria; 3 Department of Paediatrics, Lagoon Hospitals, Apapa, Lagos, Nigeria; 4 Department of Paediatrics, National Hospital, Abuja, Nigeria; 5 Department of Child Health, College of Medical Sciences, University of Benin, Benin City, Nigeria

**Keywords:** Newborn health, Global health, Developing country, Health priorities

## Abstract

**Background:**

An understanding of the perception of paediatricians as key stakeholders in child healthcare delivery and the degree of congruence with current investment priorities is crucial in accelerating progress towards the attainment of global targets for child survival and overall health in developing countries. This study therefore elicited the views of paediatricians on current global priorities for newborn health in Nigeria as possible guide for policy makers.

**Methods:**

Paediatric consultants and residents in the country were surveyed nationally between February and March 2011 using a questionnaire requiring the ranking of nine prominent and other neonatal conditions based separately on hospital admissions, mortality, morbidity and disability as well as based on all health indices in order of importance or disease burden. Responses were analysed with Friedman test and differences between subgroups of respondents with Mann-Whitney *U* test.

**Results:**

Valid responses were received from 152 (65.8%) of 231 eligible physicians. Preterm birth/low birthweight ranked highest by all measures except for birth asphyxia which ranked highest for disability. Neonatal jaundice ranked next to sepsis by all measures except for disability and above tetanus except mortality. Preterm birth/low birthweight, birth asphyxia, sepsis, jaundice and meningitis ranked highest by composite measures while jaundice had comparable rating with sepsis. Birth trauma was most frequently cited under other unspecified conditions. There were no significant differences in ranking between consultants and residents except for birth asphyxia in relation to hospital admissions and morbidity as well as sepsis and tetanus in relation to mortality.

**Conclusions:**

Current global priorities for neonatal survival in Nigeria largely accord with paediatricians’ views except for neonatal jaundice which is commonly subsumed under “other“ or "miscellaneous" neonatal conditions. While the importance of these priority conditions extends beyond mortality thus suggesting the need for a broader conceptualisation of newborn health to reflect the current realities, paediatricians should be actively engaged in advancing the attainment of global priorities for child survival and health in this population.

## Background

Despite declining under-5 mortality rates worldwide, recent global estimates still suggest that about 8.8million children die every year out of which 41% (about 3.6million deaths) occur in the neonatal period compared with 37% a decade ago [1]. Similarly, while neonatal mortality in Nigeria declined from 53 to 39 per 1000 live births in the last decade, the country still accounts for 8.3% of global neonatal deaths (behind India and China). Based on the annual rate of change from 1990 to 2011, Nigeria is one of the 23 countries in sub-Saharan Africa that are unlikely before 2040 to achieve the Millennium Development Goal (MDG) 4 of reducing the under-5 mortality rate by two-thirds between 1990 and 2015 [2]. Complications of preterm birth, birth asphyxia, infections, diarrhoea, tetanus and congenital abnormalities have been extensively reported as the leading causes of neonatal deaths for which priority investment is urgently required to build requisite national capacity to effectively address these conditions [1].

Besides the prevailing concerns about the slow pace of progress on mortality reduction in some developing countries, morbidity and disability are gaining recognition also as important indices of newborn health especially within the context of the expanding populations of child survivors [3,4]. For example, preterm birth, birth asphyxia and congenital abnormalities are not only the leading causes of mortality but also associated with significant morbidity and long-term sequelae in survivors [5,6]. However, the relative importance and contributions of these conditions to neonatal hospitalisation, morbidity and disability remain unknown and thus difficult to prioritise due to lack of population-based data. Survey of physicians has always served as a valuable tool and cost-effective source of information in health services and policy research worldwide especially in resource-limited settings [7]. The critical role of physicians in influencing individual and community health-seeking behaviour is also widely acknowledged thus making them valuable partners in public health promotion. Additionally, it is more likely that paediatricians would demonstrate a higher level of commitment to policy initiatives that reflect the views of the majority and respected colleagues in comparable circumstances as far as practicable [8]. This study therefore set out to establish the views of paediatricians on priorities for newborn survival and health and the relationship with current global health priorities in Nigeria.

## Methods

We surveyed paediatric consultants and residents on the electronic mailing list of the Paediatric Association of Nigeria (PAN) as at 31^st^ December 2010. Four of the authors verified the list of eligible recipients as the PAN mailing list also included non-paediatricians that had attended its past conferences. Respondents were requested to complete either an online version (Survey Monkey; Palo Alto, CA, USA) or a one-page MS Word version of a questionnaire ( Additional file 1). The questionnaire was pretested for clarity, comprehension and completion time among eight paediatricians in different public and private hospital settings in Nigeria as well as one US-based neonatologist with extensive experience with the local practice since 1994. The first invitation was made in February, 2011 and continued till end of March 2011. The study rationale was communicated in a covering letter and this was followed with repeated appeals for participation. Those who were missed during the first invitation as identified by the authors were further contacted prior to the analysis of the data. No financial incentives were offered to prospective respondents. As this study was essentially an audit of professional opinion and no definite data on human subjects was solicited, formal institutional approval as stipulated by the Helsinki Declaration was not considered mandatory.

Basic demographic and work-related personal profile was requested from each respondent. This included sex, age, years in practice since primary medical qualification and membership status with PAN. Respondents were also required to state their position/status in current, most recent or last appointment (Professor, Lecturer, Consultant, Resident or Medical Officer), the type of practice setting (Tertiary, General or Secondary, Health Centre, Other) and type of employer (Government or Private Ownership, Non-Governmental Organisation, International Organisation or Other). Each respondent was then required to rank nine specific neonatal conditions in order of importance or disease burden based on their contributions to four key health outcomes. The selected conditions were preterm birth/low birth weight, birth asphyxia, sepsis, pneumonia and meningitis, diarrhoea, tetanus, jaundice and congenital abnormalities based on the International Classification of Diseases, 10^th^ revision (ICD-10) and relevant literature [1,3]. A tenth category was created for other unspecified conditions. Jaundice, usually subsumed under “other neonatal conditions” was identified separately because of available regional/local evidence on its contribution to all four outcomes: hospital admissions for special care, mortality, morbidity and disability [5,9-15]. We hypothesised that the practitioners’ overall priorities will be reflected in the pattern of allocating financial resources among the various conditions with a given budget. Respondents were therefore requested to indicate how they would allocate a hypothetical grant of US$100,000 across these conditions as a measure of the composite priority across the four main outcomes.

### Statistical analysis

The responses were analysed using IBM SPSS Statistics (Version 20). The individual rankings were rated in reverse order with the most preferred rated as 10. The mean ranks for the 10 conditions across the four outcomes and the financial allocation by respondents were determined with Friedman non-parametric test as no assumptions were made on the underlying distribution of the data. Perceived differences between specific groups of respondents for each of the health measures were explored with a two-tailed Mann-Whitney *U* test. Statistical significance was assessed at critical level of p <0.05.

## Results

A total of 73 online and 94 paper responses were received. Two online duplicates, one invalid online response, five paper duplicates of online responses and seven ineligible paper responses from medical or house officers were excluded. A total of 152 valid responses representing 65.8% of the 231 eligible respondents from our mailing list were analysed. The respondents were 15 professors, 77 consultants and 60 residents spread over half (22) of the 36 States and Federal Capital Territory (Table 1). Overall, responses were received from 17 (85.0%) of the 20 States with hospitals accredited for postgraduate training in paediatrics.

**Table 1:** Characteristics of respondents (n = 152)

**Factor**	**Frequency**
Sex	
Female	72 (47.4%)
Male	80 (52.6%)
Age	
Median	41 years
Interquartile range	35 – 48 years
Post-MBBS experience	
Median	17.4 years
Interquartile range	9 – 25 years
Status	
Professor	15 (9.9%)
(Senior) Lecturer/Consultant	77 (50.7%)
Paediatric resident	60 (39.4%)
Practice setting	
Tertiary/Teaching hospital	124 (81.6%)
General/Secondary hospital	22 (14.5%)
Private clinic	6 (3.9%)
Employers	
Federal/State government	136 (89.5%)
Private organisation	15 (9.8%)
International	1 (0.7%)
Geographical setting	
North (11 States + Federal capital territory)	43 (28.3%)
South (10 States)	109 (71.7%)

Summary rating scores by participants showing the descriptive statistics are presented in Table 2. The Friedman tests showed statistically significant differences in the median scores across all neonatal conditions for hospital admissions (p <0.001), mortality (p <0.001), morbidity (p <0.001) and disability (p <0.001). Post-hoc tests of significance for pair-wise comparison of all ten conditions for each of the five health outcomes were not explored as these were not considered critical to the overall objective of the study and in view of the sheer number of possible combinations.

**Table 2:** Summary of rating scores by all participants for all neonatal conditions

**Conditions**	**Median (Interquartile Range); Mean Rank**			
	**Admissions**	**Mortality**	**Morbidity**	**Disability**
Prematurity/LBW	9.0 (8.0–10.0); 8.9	9.0 (8.0–10.0); 8.9	9.0 (8.0–10.0); 8.8	8.0 (7.0–9.0); 7.9
Birth asphyxia	9.0 (7.8–9.3); 8.4	9.0 (8.0–10.0); 8.4	9.0 (8.0–10.0); 8.5	10.0 (9.0–10.0); 9.0
Sepsis	8.0 (7.0–9.0); 8.3	7.0 (6.0–8.0); 6.9	7.0 (5.0–8.0); 6.9	5.0 (4.0–6.0); 5.5
Meningitis	5.0 (4.0–6.0); 5.2	6.0 (5.0–7.0); 6.0	6.0 (5.0–7.0); 6.0	7.0 (6.0–8.0); 7.2
Pneumonia	5.0 (4.0–5.3); 4.5	4.0 (3.0–6.0); 4.5	4.0 (3.0–5.0); 4.4	4.0 (3.0–4.3); 3.8
Diarrhoea	3.0 (2.0–4.0); 3.3	3.0 (2.0–4.0); 3.0	3.0 (2.0–4.0); 3.1	2.0 (2.0–3.0); 2.5
Tetanus	4.0 (3.0–5.0); 4.1	7.0 (4.0–8.0); 6.2	5.0 (3.0–6.0); 4.7	4.0 (2.0–6.0); 4.2
Jaundice	7.0 (7.0–9.0); 7.4	5.0 (3.0–6.0); 4.9	7.0 (6.0–8.3); 6.8	8.0 (6.8–9.0); 7.6
Congenital defects	3.0 (2.0–5.0); 3.4	4.0 (2.0–6.0); 4.6	4.0 (2.0–6.0); 4.4	6.0 (4.0–8.0); 5.9
Others	1.0 (1.0–1.3); 1.6	1.0 (1.0–1.0); 1.6	1.0 (1.0–1.0); 1.5	1.0 (1.0–1.0); 1.5
Friedman test	p <0.001	p <0.001	p <0.001	p <0.001

**Figure 1:**
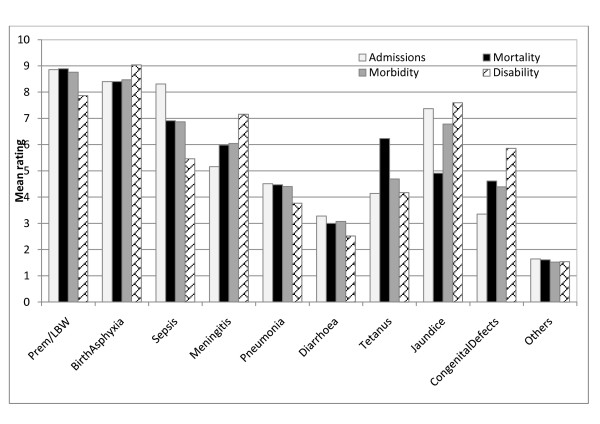
Comparison of priority ratings of indices of newborn health.

A graphical presentation comparing the mean rank scores for all conditions across the four health indices is shown in Figure 1. Except for disability, preterm birth/low birth weight ranked highest by all measures while birth asphyxia ranked second highest but highest for disability. Sepsis ranked above jaundice on hospital admissions, mortality and morbidity, but lower for disability. Jaundice ranked next to preterm birth/low birth weight and birth asphyxia for disability; and above tetanus by all measures except mortality. Similarly, meningitis ranked above tetanus by all measures except disability while diarrhoea ranked the least of all specific conditions by all measures.

The subgroup analysis for hospital admissions showed no significant differences between professors and consultants for all neonatal conditions. However, birth asphyxia was ranked significantly lower by residents compared with professors (p = 0.023) or consultants (p = 0.026). In relation to mortality, sepsis was ranked significantly lower by residents compared with either professors (p = 0.038) or consultants (p = 0.038) while tetanus was ranked significantly higher compared with professors (p = 0.022) or consultants (p = 0.010). No differences were found between professors and consultants across all conditions. Based on morbidity, birth asphyxia was ranked significantly lower by residents compared with consultants (p < 0.001). No significant differences were observed between subgroups for all specific conditions in terms of disability and budget allocation.

**Figure 2:**
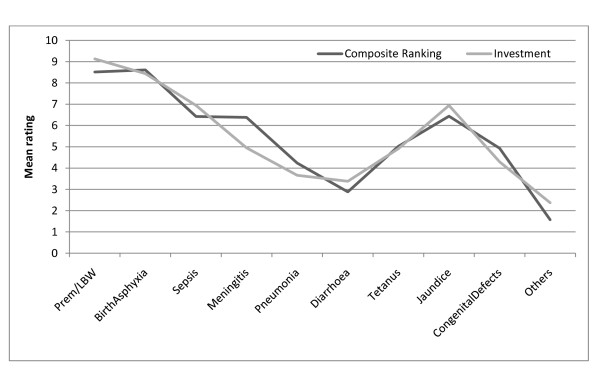
Comparison of priority ratings based on composite ranking all conditions and planned investment.

The average or composite ratings across all measures compared with the priorities for investment are shown in Figure 2. As hypothesised, there was concordance between the composite ratings and investment profile. The top five priorities (mean rank ≥5) were preterm birth/low birth weight, birth asphyxia, sepsis, jaundice and meningitis. Jaundice ranked pari-passu with sepsis by composite rating and level of proposed investment.

Conditions typically mentioned under “others” included umbilical cord bleeding, meconium aspiration, macrosomia, congenital heart disease, birth injuries traditional medications, severe anaemia, HIV and metabolic disorders. Only 39 respondents made free remarks which included “separating septicaemia with/without non-pneumonia respiratory disorder from pneumonia might be rather challenging”, “neonatal sepsis and meningitis could be taken together since the usual practice is to exclude or confirm meningitis once sepsis is suspected”, “we seldom see cases of neonatal tetanus” and “degree of ignorance about neonatal jaundice even among the educated is alarming”.

## Discussion

This survey suggests that paediatricians’ views accord with various published reports that rank preterm birth/low birth weight, birth asphyxia and sepsis as the leading causes of neonatal mortality in Nigeria [1,16,17]. This is reassuring as recent reports suggest that most countries in Sub-Saharan Africa are unlikely to achieve neonatal mortality rates comparable to levels currently reported in high-income countries before 2065 at the existing rate of progress [18]. Efforts to accelerate reduction in neonatal mortality rate such as promotion of delivery with skilled birth attendants, the ‘helping babies breathe’ global initiative for neonatal resuscitation and revision of the integrated management of childhood illness (IMCI) algorithms to improve management of neonatal infections and preterm births therefore deserve greater attention from all stakeholders at all levels of health care delivery.

However, paediatricians’ views on these priority conditions are not limited to mortality and underscore the need not to overlook the full health spectrum of these conditions especially as the vast majority of infants are delivered outside hospitals. For example, the economic burden of preterm births/low birth weight survivors in terms of immediate hospital care and long-term support are beyond the means of most families [19]. While the prevention of preterm births altogether remains unattainable goal worldwide, the health care system even at the tertiary level is generally ill-equipped to provide on-going care for otherwise healthy children with special needs. An integrated approach for the management of preterm/low birth weight infants is therefore essential in effectively addressing the associated immediate and long-term burden.

Perhaps the most notable finding is the comparable overall rating for sepsis and jaundice after preterm births and birth asphyxia. Evidently, the common practice of subsuming neonatal jaundice under “other conditions” in various reports on global child health seems inappropriate for Nigeria and perhaps possibly for other countries in Africa where severe jaundice has been associated with significant morbidity and mortality [5,9,10,12,17]. Available facilities in many hospitals make it impracticable to accurately distinguish between early-onset sepsis and jaundice based on the immediate clinical signs or symptoms [20,21]. It is therefore not uncommon for infants with jaundice to be treated routinely for suspected sepsis as a first-line intervention until laboratory investigations confirm otherwise even though both conditions are more often unrelated in this setting.

The adverse consequences of severe jaundice and acute bilirubin encephalopathy are entirely preventable through effective clinical and surveillance protocol. Close and objective monitoring of bilirubin levels as well as prompt treatment with phototherapy is all that is needed by majority of the affected infants failing which exchange transfusion is warranted. However, the benefits of these treatments are ‐ seldom available due to the lack of requisite or functional facilities. Early hospital discharge within 48 hours of birth or delivery outside hospital often results in late presentation in hospitals. This delay is often exacerbated by poor recognition of jaundice especially by first-time mothers and the common recourse to traditional herbal therapies before seeking medical attention [22]. Even in hospitals with phototherapy units, lack of routine maintenance and evaluation of the irradiance levels often results in high rates of exchange transfusions [11,23]. The only community-based data on severe neonatal jaundice in Nigeria suggests an incidence of 55 per 1000 infants requiring phototherapy and 19 per 1000 infants requiring exchange blood transfusion [24]. Widespread glucose-6-phosphate dehydrogenase (G6PD) deficiency is also a principal predisposing factor to severe jaundice in Nigerian infants aggravated by (TA)_n_ promoter polymorphism of the uridine-diphospate-glucuronosyltransferase 1A1 gene (UGT1A1) [25] and possibly the active promotion of exclusive breastfeeding [26]. Appropriate policy initiatives embracing maternal and health professional education, provision of functional phototherapy units and bilirubin monitoring devices are evidently warranted to prevent or significantly reduce the unrecognised contributions of jaundice to neonatal mortality in Nigeria as well as the related morbidity and disability among the survivors.

While tetanus remains as a significant cause of mortality, the overall ranking below jaundice may reflect both global and national progress in curtailing its incidence largely due to improved routine tetanus toxoid immunisation and greater awareness on the importance of clean cord care in hospitals and among primary health care workers including traditional birth attendants [1,27-30]. Nonetheless, concerns still persist on the unacceptably high levels of tetanus-related mortality in many locations which deserve urgent attention. Traditional uvulectomy is the suspected portal of entry in majority of cases followed by the unhealed umbilical cord. The traditional practice of cutting the uvula between the third and seventh days of life as well as unhygienic handling of the umbilical cord are major contributory factors to the high incidence of neonatal tetanus. Current global efforts aimed at promoting facility-based delivery or home-delivery attended by trained midwives as well as clean birth and postnatal practices as recommended by the World Health Organisation: and immunisation of pregnant women and those of childbearing age obviously need to be intensified at all levels of obstetric/perinatal care delivery.

It is not uncommon for the term “neonatal sepsis” to be collectively used for septicaemia, meningitis and pneumonia because of the challenge of diagnosing these infections accurately in many resource-poor settings and lack of uniform clinical criteria for their evaluation. This fact is worth noting in interpreting our results. As expected, the relative importance of congenital abnormalities as a cause of neonatal deaths diminishes as other largely preventable causes of death remain prominent in contrast to the pattern in countries with well-established healthcare systems. Notwithstanding, the disability associated with this condition was rated higher than the related mortality. The ranking for diarrhoea accurately suggests a far lesser burden among neonates compared with older infants and young children worldwide. For example, diarrhoea accounts for 18% of child deaths in Africa compared to 1% of neonatal deaths [1]. Birth trauma was most widely cited in the category of “other conditions” which perhaps reflects on the quality and challenges of obstetric care delivered in some hospitals especially at the secondary levels.

The lower ranking for birth asphyxia in relation to hospital admissions, mortality and morbidity among residents compared with consultants warrant further investigation. Similarly, it was unclear why residents compared with consultants ranked tetanus higher and sepsis lower in terms of mortality. The views of residents as first line physicians must be balanced by cumulative experience of consultants and professors in explaining the observed differences. The lack of differences between professors and consultants should be expected as the professors are themselves consultants in tertiary clinical settings with academic distinctions.

The major strengths of this study are its novelty as well as the geographical representativeness and working experience of respondents besides the prospect of facilitating a mutually-shared awareness between policy makers and paediatricians on the priorities for newborn health in this population. The response rate would also appear satisfactory considering prevailing challenges to web-based surveys especially among busy physicians in settings with limited internet connectivity. While the lack of comprehensive demographic data on those contacted precluded comparison of respondents and non-respondents, a random interview of non-respondents for example, suggested that some individuals did not consider it necessary to participate once they ascertained that their colleagues more closely associated with neonatal care had responded resulting in some response bias. It was also not unlikely that some eligible respondents were not enlisted with PAN or were wrongly excluded during verification with potential for selection bias. Notwithstanding, the key findings accord with available literature from Africa on neonatal health and support calls to also pay attention to the optimal growth and development of the many survivors of these conditions as far as practicable. Perhaps more importantly, this study exemplifies a practical approach to overcoming the constraints of requisite data drought on some aspects of newborn health in resource-poor countries.

## Conclusion

While current global priorities for neonatal survival in Nigeria are derived from limited published data the evidence from this survey suggests that they largely accord with paediatricians’ views except for neonatal jaundice which is commonly subsumed under miscellaneous neonatal conditions. The need to recognise the importance of these priority conditions beyond mortality is also demonstrated. These findings should motivate paediatricians to play a more active role in advancing appropriate interventions to facilitate the attainment of current performance targets for newborn health in this population.

## 

This work has been presented at the 5^th^Scientific Conference of the Nigerian Society for Neonatal Medicine (NISONM), Lagos, Nigeria, June 27-29, 2012.

## Competing interests

The authors declare that they have no competing interests.

## Authors’ contributions

BOO conceived and designed the study with critical inputs from EKA. BOO, EKA and CVE participated in data collection from Southwest Nigeria, GEO from Southeast Nigeria and MMY from Northern Nigeria. All authors contributed to the data analysis and the interpretation of the results. BOO drafted the manuscript. All other authors critically reviewed the draft, read and approved the final manuscript.

## Pre-publication history

The pre-publication history for this paper can be accessed here:

http://www.biomedcentral.com/1472-698X/12/9/prepub

## Supplementary Material

Additional file 1 Survey on neonatal care in Nigeria. Click here for file
