# Supplementary material for: Paediatricians’ perspectives on global health priorities for newborn care in a developing country: a national survey from Nigeria
Source: BMC Int Health Hum Rights. 2012 Jul 2;12:9. doi: 10.1186/1472-698X-12-9 (PMC3519520; doi:10.1186/1472-698X-12-9)
Supplement: Additional file 1 — Survey on neonatal care in Nigeria. [file 1472-698X-12-9-S1.pdf]

SURVEY ON NEONATAL CARE IN NIGERIA

RESPONDENT PROFILE: (Check or type as applicable)

SEX: M ☐ F ☐ AGE (YRS): YEARS POST-MBBS: PAN MEMBER: Y ☐ N ☐

CURRENT POSITION (Check as applicable):

PROFESSOR: ☐ (Senior) LECTURER: ☐ CONSULTANT: ☐ RESIDENT: ☐ MEDICAL OFFICER:☐

CURRENT/MOST RECENT PRACTICE SETTING (Check as applicable):

TERTIARY: ☐ GENERAL/SECONDARY: ☐ HEALTH CENTRE: ☐ OTHER: ☐

EMPLOYER(S) (Check as applicable):

GOVERNMENT: ☐ PRIVATE: ☐ NGO: ☐ INTERNATIONAL: ☐ OTHER: ☐

INSTRUCTIONS

The following conditions are associated with significant neonatal mortality, morbidity and long-term disability in Nigeria. Kindly rank these conditions on a priority/burden scale of 1 (highest) to 10 (least) for the specified criteria in columns A, B, C & D based on your current or past experience in newborn care. In addition, please indicate in the last column (E) how you may allocate a possible grant of US$100,000 across these conditions to address the burden of disease in your setting.

CONDITIONS	A<br>**HOSPITAL ADMISSIONS**	B<br>**MORTALITY**	C<br>**MORBIDITY**	D<br>**DISABILITY**	E<br>**ALLOCATION US$**
**PREMATURITY/LBW**					
**BIRTH ASPHYXIA**					
**NEONATAL SEPSIS**					
**NEONATAL MENINGITIS**					
**NEONATAL PNEUMONIA**					
**DIARRHOEA**					
**NEONATAL TETANUS**					
**NEONATAL JAUNDICE**					
**CONGENITAL DEFECTS**					
**OTHERS** (Specify below)*****					
*				**TOTAL US$**	**100,000.00**

REMARKS (IF ANY):

EMAIL: DATE:

© Healthy Start Initiative
